# Vaccine Development Throughout History

**DOI:** 10.7759/cureus.16635

**Published:** 2021-07-26

**Authors:** Amr Saleh, Shahraz Qamar, Aysun Tekin, Romil Singh, Rahul Kashyap

**Affiliations:** 1 Faculty of Medicine, Mansoura University, Mansoura, EGY; 2 Post-baccalaureate Research Education Program, Mayo Clinic, Rochester, USA; 3 Critical Care, Mayo Clinic, Rochester, USA

**Keywords:** vaccine development, infectious and parasitic diseases, smallpox, polio, rabies, cholera, covid-19

## Abstract

The emergence of the coronavirus disease 2019 (COVID-19) pandemic has made us appreciate how important it is to quickly develop treatments and save lives. The race to develop a vaccine for this novel coronavirus began as soon as the pandemic emerged. Time was the only limiting factor. From the first vaccine developed in 1796 against smallpox to the latest COVID-19 vaccine, there have been several vaccines that have reduced the burden of disease, with the associated mortality and morbidity. Over the years we have seen many new advancements in organism isolation, cell culture, whole-genome sequencing, and recombinant nuclear techniques. These techniques have greatly facilitated the development of vaccines. Each vaccine has its own development story and there is much wisdom to be gained from learning about breakthroughs in vaccine development.

## Introduction and background

**Figure 1:**
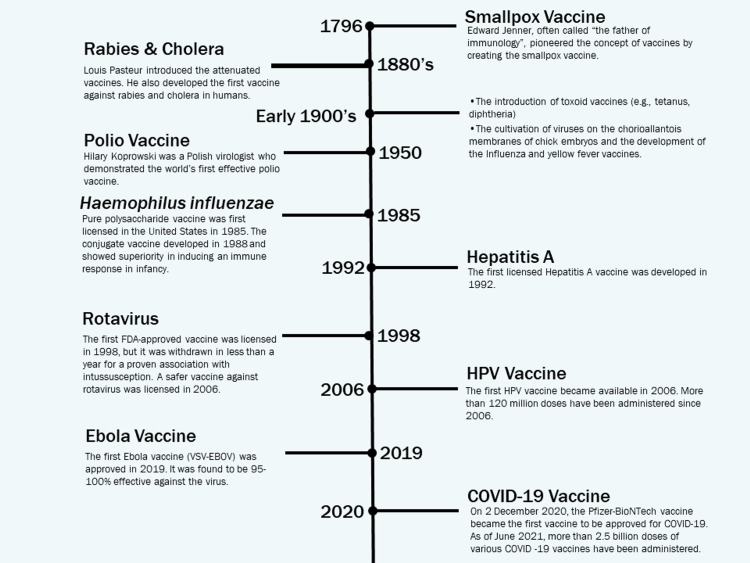
Vaccine history timeline. COVID-19, coronavirus disease 2019; FDA, U.S. Food and Drug Administration; HPV, human papillomavirus

Although inoculation practices were started more than 500 years ago, the term vaccine was first described in the 18th century by Edward Jenner. It is derived from Vacca, a Latin word for cow. Jenner inoculated an eight-year-old boy with cowpox lesions from the hands of milkmaids in 1796. This ultimately conferred immunity against smallpox. After 80 years, Louis Pasteur was instrumental in developing a live attenuated vaccine against rabies in humans which was highly successful. In the 19th century, we witnessed the evolution of germ theory through the discovery of numerous microorganisms by Koch. By the mid-20th century, after the introduction of attenuated toxins (toxoids) the first generation of vaccines were developed. Through this development, it was possible to make vaccines for diphtheria and tetanus. In the 1930s, major advances in lab techniques allowed the cultivation of viruses on the chorioallantoic membranes of chick embryos. This led to the development of influenza and yellow fever vaccines. The evolution of cell culture 15 years later led to the creation of the polio vaccine, and this marked the beginning of the golden age of vaccines. During this period a series of important vaccines like the measles, mumps, rubella, and varicella vaccines were developed. The introduction of recombinant DNA and whole-genome sequencing techniques were major milestones in vaccine development. It gave researchers the tools to develop new vaccines against pathogens, which was not possible before. We aim to cover the timeline and development of most of the vaccines developed during the last century (Figure 1) [1-3].

## Review

Smallpox

Smallpox is one of the oldest known infectious diseases. It has caused hundreds of millions of deaths. The earliest written records of this disease go back to China in the 4th century. Some studies postulate that there were smallpox-like rashes found on Egyptian mummies suggesting that it may even go back at least 3000 years [4]. In 1980, World Health Organization (WHO) declared smallpox to be the first disease to be eradicated worldwide because of aggressive immunization efforts.

The earliest documented trials of variolation were in China and India during the 16th century. This was achieved by the inoculation of smallpox pus or scabs either by a nasal or cutaneous route. During the smallpox outbreak worldwide, early inoculation trials were carried out in Britain and colonial Massachusetts in 1721. Also, there had been reports of inoculations from the Ottoman Empire and North Africa. Although the origins of inoculation are not definite, whether it arose from Asia or Africa, the practice of vaccination was first introduced by Edward Jenner in 1796 [5]. He observed that the milkmaids who had cowpox lesions were immune against smallpox infection. He then exposed a young boy to the milkmaids’ lesions and observed that the boy got immunity against smallpox. He extended this practice to more children and similar outcomes were recorded [6]. Since then, there have been continuous efforts to develop safer vaccine techniques.

Currently, three main vaccines for smallpox are approved by FDA. Dryvax® was one of the main combinations used to eradicate smallpox in the early 20th century. Its production started in the 19th century in Wyeth Laboratories and was suspended in 1982 after global eradication was achieved. Dryvax® remained in stockpile till 2008 until it was replaced by ACAM2000, a second-generation vaccine with a safer, yet with the same efficacy profile [7]. The most recent smallpox vaccine is MVA-BN. It was approved in Europe and Canada in 2013 and the USA in 2019. MVA-BN has a better safety profile in comparison with Dryvax® and ACAM2000 in patients with atopic dermatitis and immunodeficiency [8]. Although smallpox was eradicated, the Healthcare Infection Control Practices Advisory Committee and Advisory Committee on Immunization Practices recommend preoutbreak measures among certain high-risk groups [9]. 

HPV

Human Papillomavirus (HPV) is the most common cause of cervical cancer, the fourth most common cancer in women worldwide [10]. HPV was first detected in cervical cancer biopsies in 1983 [11]. Two decades later the earliest publication on the first HPV vaccine clinical trials was released. The first HPV vaccine was approved by the FDA in 2006 [10, 12]. Universal vaccination programs against HPV have resulted in a significant reduction in cervical cancer incidence and mortality. 

Ebola viruses

After the first Ebola virus disease epidemic was documented in 1976 in West Africa, multiple short-term explosive outbreaks occurred with variable severity. The deadliest and latest outbreak was in 2014 with a case fatality rate of up to 85%. This led the WHO to declare the epidemic as an international public health emergency in August 2014 [13-14]. Before that, multiple non-human trials were underway to develop a vaccine against the Ebola virus family [15]. In 2015, the race to create a safe and effective vaccine began, and many human clinical trials were established to test multiple vaccine candidates [16].

For rapid safety and immunogenicity testing, a replication-competent recombinant vesicular stomatitis virus (rVSV)-based vaccine expressing a Zaire ebolavirus (ZEBOV) glycoprotein was used [16]. Under the brand name Ervebo®, it was authorized by the European Medicines Agency (EMA) in Nov 2019 and by the FDA in Dec 2019 [17-18]. The second vaccine is the two-dose heterologous Ad26.ZEBOV and MVA-BN-Filo Ebola vaccine regimens, which are two different vaccines given about 56 days apart. It was approved for medical use by EMA in July 2020 [14, 19-20]. While many vaccines are still under trial, those are the only approved vaccines against the Ebola family of viruses.

Rotavirus

Rotavirus was first discovered in 1973 and was found to be the major causative agent of acute gastroenteritis in childhood. Several clinical trials phase I-III have been conducted since 1981 to develop a safe vaccine against Rotavirus. The first FDA-approved vaccine was licensed under the brand name Rotashield® in 1998, but it was withdrawn in less than a year because of a proven association with intussusception. It took another eight years, till a safer vaccine against rotavirus was licensed. In 2006, RotaTeq® (RV5: Pentavalent) was introduced into the universal vaccination program in the USA. Then, Rotarix® (RV1: Monovalent) was licensed by FDA in 2008 [21]. A meta-analysis of multiple case-control studies in the United States, comparing the effectiveness of different rotavirus vaccine types, did not show a statistically significant difference [5]. 

Hepatitis A virus

The Hepatitis A virus was first detected in the United States in 1973. It took three decades to develop a safe and effective vaccine against it. The first vaccine against Hepatitis A was called Havrix® and was licensed in 1992. This was followed by the vaccine Vaqta® which was licensed in 1993. Those are the only licensed vaccines against the Hepatitis A virus in the United States [22-23].

Haemophilus influenzae

Haemophilus influenzae was first described in 1892. Initially, it was mistakenly considered as the cause of Influenza pandemics, until in 1933 when the viral etiology was revealed [24-25]. It is responsible for a wide range of localized and invasive infectious diseases. It is classified into capsulated and nonencapsulated variants. The capsulated was subclassified into six serotypes a-f according to the capsular antigens. The most virulent and invasive serotype is the type b strain (Hib). The first vaccine developed against Hib was in the early 1970s in Finland. It was composed of the capsular polysaccharide capsule polyribosylribitol phosphate (PRP). Pure polysaccharide vaccines were first licensed in the United States in 1985 and recommended for those over 18 months. Studies showed no efficacy of PRP vaccines in ages below 18 months due to the immaturity of the immune system. It was used until 1988 when it was shown that conjugate vaccines are better in inducing an immune response in infancy which is considered the peak age for invasive Hib infections. The PRP attachment to a protein conjugate as tetanus and diphtheria toxoids was the initial step for developing conjugate vaccines. Currently, the licensed products are combinations with other vaccine products such as Hepatitis B and Diphtheria Pertussis Tetanus vaccines [26].

Influenza

The exact timing of influenza pandemics is not known exactly. The earliest Influenza-like illness was mentioned by Hippocrates in 400 BC, in the “Book of Epidemics”, however, not all researchers think that this was actually influenza. The first pandemic that certainly matched the current description of influenza was documented in 1580 [27]. Since then, multiple influenza pandemics have occurred throughout the world. One of the most devastating pandemics was the “Spanish flu” in 1918 which caused tens of millions of deaths. It was mistakenly thought that the pandemic had a bacterial etiology, until 1931 when the influenza virus was isolated from nasal secretions of infected patients. The first trials to develop a vaccine were in the mid-1930s. In 1938 a successful experiment to develop a monovalent inactivated vaccine was conducted to protect the US military against the Influenza A virus. The vaccine was approved for public use in the United States in 1945. Soon a new strain “Influenza B” was discovered, and a few years later the first bivalent vaccine was licensed for public use [27].

The antigenic shifts and drifts and continuous changes in the virus compositions necessitated the establishment of a surveillance system for the circulating influenza strains. The first surveillance was created by the WHO in 1952. The discovery of newer strains and the different types of hemagglutinin and neuraminidase antigens in different pandemics all over the world lead to the introduction of the Trivalent vaccine in the 1970s. Subsequently, the Quadrivalent vaccine was licensed by the FDA in 2012. Advancements in cell culture techniques, recombinant DNA, and whole genome sequencing gave scientists the ability to rapidly respond to the evolving Influenza pandemics like the “Avian flu” in 1997 and the “Swine flu” in 2009, by creating safe and effective vaccines within a few months. Current research priorities include the development of universal vaccines that can respond to the current and evolving strains [27].

COVID-19

The first reported cases of COVID-19 were in Dec 2019 in Wuhan, China. A cluster of patients with unexplained respiratory infections was shown to be caused by a novel coronavirus named SARS-CoV-2 [28-30]. This virus belongs to the Coronaviridae family which also includes the severe acute respiratory syndrome coronavirus-1 (SARS-CoV-1) and Middle East respiratory syndrome coronavirus (MERS-CoV). COVID-19 was declared as a pandemic by WHO on March 11th, 2020 [31-32]. Fever, myalgia, cough, dyspnea, and flu-like symptoms are the most frequent symptoms of COVID-19, which predominantly affects the respiratory system. From asymptomatic infection to respiratory failure, multi-organ dysfunction, and death, the COVID-19 has a wide spectrum of clinical presentations [33]. COVID-19 has been linked to harmful effects on other body systems in addition to the respiratory system [34-38]. Although SARS-CoV-2 has a lower mortality rate in comparison with SARS-CoV and MERS-CoV, the transmissibility and spread are much higher than SARS-CoV-1 and MERS. In early 2020, the race for developing an effective and safe vaccine began. Soon there were more than 200 candidates in preclinical and clinical development all over the world. A fewer number reached phase III of the clinical trials, and in Dec 2020 the first COVID-19 vaccine, created through a collaboration of Pfizer and BioNTech, was approved. COVID-19 vaccine development is considered one of the fastest in the history of vaccine science. SARS-CoV-1 and MERS vaccine candidates did not go beyond phase I because of the limited spread of the disease and diminished demand, but they helped to understand how the body reacts to coronaviruses [39]. On Dec 31st, 2020 the WHO issued an Emergency Use Listing (EULs) for the Pfizer COVID-19 vaccine (mRNA vaccine). On Feb 15th, 2021 the WHO issued a EUL for the AstraZeneca/Oxford COVID-19 vaccine (Adenovirus vector vaccine). On Mar 12th, 2021 the WHO issued a EUL for the Johnson & Johnson COVID-19 vaccine (Adenovirus vector vaccine). Currently, those are the only vaccines issued by the EULs. Another mRNA vaccine manufactured by Moderna was licensed by FDA on Dec 18th, 2020, and the EMA on Jan 6th, 2021. While other vaccines are being distributed in other countries like Sinopharm, Coronavac, Novavax, and Sputnik vaccines, they are not included in the Emergency Use Listing issued by the WHO at the time of writing this article [40-41].

Different types of vaccines, their administration route, and their mechanism of action are summarized in Table 1.

**Table 1:** A summary of different types of vaccines. *COVID-19 vaccine license dates are provided based on WHO recommendations. IM, intramuscular; HPV, human papillomavirus

Disease agent	Year identified	Vaccine name	Year licensed (FDA)	Vaccine type	Route of administration
Smallpox	300 AD	Vaccine by Edward Jenner	1796	Extract of cowpox lesions on milkmaids’ hands.	Skin punctures
		Dryvax	Late 19^th^ century (withdrawn)	Lyophilized, live-virus preparation	Bifurcated needle punctures
		ACAM2000	2007	Cell cultured, Live virus	Bifurcated needle punctures
		MVA-BN	2013	Recombinant	IM
Haemophilus influenza	1933	Hib	1985 (withdrawn)	Capsular polysaccharide capsule polyribosylribitol phosphate	IM
		PedvaxHIB	1990	Meningococcal protein conjugate	IM
		ActHIB	1993	Tetanus toxoid conjugate	IM
		Hiberix	2009	Tetanus toxoid conjugate	IM
Influenza	1933	1^st^ Bivalent	1945	Inactivated	IM
		1^st^ Trivalent	1978	Inactivated	IM
		FluMist	2004	Live attenuated vaccine	Intranasal
		1^st^ Quadrivalent	2012	Inactivated	IM
Hepatitis A virus	1973	Havrix	1992	Inactivated	IM
		VAQTA	1993	Inactivated	IM
Rotavirus	1973	Rotashield	1998 (withdrawn)		
		RotaTeq	2006	Live virus	Oral
		ROTARIX	2008	Live virus	Oral
Ebola virus	1976	ERVEBO	2019	Live virus	IM
HPV	1983	Gardasil	2006	Recombinant	IM
COVID-19*	2019	Pfizer-BioNTech	Dec 2020	mRNA vaccine	IM
		AstraZeneca/Oxford	Feb 2021	Adenovirus vector	IM
		Covishield	Feb 2021	Adenovirus vector	IM
		Johnson & Johnson	Mar 2021	Adenovirus vector	IM
		Moderna	April 2021	mRNA vaccine	IM
		Sinopharm-BBIBP	May 2021	Inactivated	IM
		CoronaVac	June 2021	Inactivated	IM

## Conclusions

Vaccines have played a crucial role in reducing the burden of infectious diseases. It all started with the initial inoculation efforts by the Chinese and the Indians 500 years ago. The journey of this practice traveled through the Ottomans and Africans landing in Europe and North America. When Edward Jenner’s ideas laid the foundation for vaccination, he hoped that his work would eventually lead to the eradication of smallpox. Since then, it has been a long journey full of challenges and failures, but his hopes were finally realized when the World Health Assembly declared the world free of this disease in 1980. The body of knowledge for developing vaccines has kept on growing, and improvements in laboratory techniques have saved millions of lives. Furthermore, the unprecedented success of COVID-19 vaccines has added another weapon in the arsenal of evidence that we have for the effectiveness of vaccines. Each vaccine has a unique developmental history and studying it can provide much-needed wisdom that might help us in future pandemics. Thus, we wanted to use this opportunity to share the results of our in-depth investigation of the development of various vaccines throughout history.
